# F93. SUBCLINICAL PSYCHOSIS COMPONENTS MAKE DIRECT AND INDIRECT CONTRIBUTIONS TO ACTIVE SUICIDE IDEATION IN ADOLESCENTS

**DOI:** 10.1093/schbul/sby017.624

**Published:** 2018-04-01

**Authors:** Richard Linscott, Theresa Parker

**Affiliations:** University of Otago

## Abstract

**Background:**

Subclinical psychosis predicts concurrent and future suicidal ideation and attempts. A key account of this relationship is that it is spurious—that suicidality and subclinical psychosis are both products of a common confounding factor such as environmental risk exposures (e.g., neglect or abuse) or the burden of general psychopathology. This account is unsatisfactory for several reasons, including that subclinical psychosis may be especially predictive of more lethal forms of suicidal behaviour. Moreover, few have considered the relationship in light of contemporary accounts of suicide. Therefore, we sought to better understand the link between subclinical psychosis and suicidality using a contemporary ideation–action framework in which perceived burden and thwarted belonging are distinguished as proximal pathways to suicidal ideation. We tested whether this framework fully mediates the relationship of subclinical psychosis with suicidal ideation, consistent with a common confounding factor account.

**Methods:**

Randomly sampled 15- to 18-year-olds from a socio-economically representative high school were invited to participate anonymously. Of those invited (n = 300), 59% provided informed consent and completed self-report measures of positive, negative, and disorganised components of subclinical psychosis (Schizotypal Personality Questionnaire [SPQ]), thwarted belonging and perceived burden (Interpersonal Needs Questionnaire), and passive and active suicidal ideation (Beck Scale for Suicide Ideation [BSS]). Participants were classified using BSS responses as non-ideators, passive ideators, or active ideators. In regression modelling (maximum likelihood estimation with bias-corrected bootstrapping), direct and indirect effects of SPQ components on ideator classifications were obtained. Mediators were perceived burden, thwarted belonging, and their interaction term. Sex and migrant status were entered as covariates.

**Results:**

Of those with complete data (n = 156), 69.9% were non-ideators, 12.8% were passive ideators, and 17.3% were active ideators. In bivariate analyses, SPQ positive scores predicted passive ideation (r = .24, p < .001) but negative (r = .13, p > .05) and disorganised scores (r = .14, p > .05) did not. In contrast, active ideation was strongly predicted by negative (r = .39, p < .001) and disorganised scores (r = .34, p < .001) and less strongly predicted by positive scores (r = .19, p < .05). Mediation models predicted passive (R2 = .29, p < .05) and active ideation (R2 = .65, p < .001). Passive ideation was sensitive only to indirect effects of SPQ scores: negative (β = .14, p < .01) and disorganised SPQ scores (β = .11, p < .05) were mediated by perceived burden. For active ideation, negative (β = .17, p < .05) and disorganised scores (β = .14, p < .05) had similar indirect effects mediated by perceived burden but there were also direct effects of positive (β = -.44, p < .01) and negative SPQ scores (β = .37, p < .05). Thwarted belonging did not mediate the effects of SPQ scores on ideator status.

**Discussion:**

A contemporary ideation–action model of suicide did not fully account for the relationship between subclinical psychosis and suicidal ideation. Instead, some components of subclinical psychosis directly influenced suicidal ideation status: Positive subclinical psychosis components protected against active suicidal ideation whereas negative components increased the risk of active ideation. Negative and disorganised components of subclinical psychosis also increased the risk of ideation by increasing the perception of self-hate and liability on others. Subclinical psychosis makes a unique contribution to the prediction of suicidal ideation.

